# Supplementary material for: New Insights into Geometric Morphometry Applied to Fish Scales for Species Identification
Source: Animals (Basel). 2024 Apr 3;14(7):1090. doi: 10.3390/ani14071090 (PMC11010809; doi:10.3390/ani14071090)
Supplement: Supplementary file 1 [file animals-14-01090-s001.zip › animals-2937539-supplementary.pdf]

## Script for the LANDMARK-BASED GEOMETRIC MORPHOMETRY

```
library(geomorph)

#loading as "data" a file .tps with the coordinates of landmarks and
semi landmarks

data<-readland.tps("coordinates.tps",specID=c("ID"))

#loading as "s" a file .csv with the indication of which points are
landmarks and which are semi landmarks

s=read.csv("matrix.csv",sep=";")
s1<-as.matrix(s)

#making the Procruste superimposition

Y<-gpagen(A=data,curves=s1)

#running the PCA analysis on the superimposed coordinates

PCA<-gm.prcomp(Y$coords)
```

## Script for the OUTLINE-BASED GEOMETRIC MORPHOMETRY

```
library("Momocs")

#importing the silohuettes

shpc <-import_jpg(auto.notcentered=TRUE, fun.notcentered=NULL,
threshold=0.5)
coo<-Out(shpc)

#loading as "file" a file .csv with the names of the species in one
column named "species"

file<-read.csv("Cartel.csv",sep=";",header=TRUE)
coo$fac<-file

#performing the Fourier analysis and the PCA

FF <-efourier(coo_scale(coo_center(coo)))
PP <-PCA(FF)
plot_PCA(PP, ~species)
```

Script for the PERMANOVA AND PAIRWISE PERMANOVA OF THE CLUSTERS IN THE PCA ANALYSES
(EXAMPLE OF THE SPECIES COMPARISON)

```
library(vegan)

#loading as "first" a file .csv with the principal components

first<-read.csv("first.csv",sep=";")

#loading as "second" a file .csv with the name of the specie in one
column named "Species" in the right order, that's the same of the
principal components

second<-read.csv("second.csv",sep=";")

#performing the PERMANOVA on the principal components using as
clusters the species

perm<-adonis2(first~Species,data=second,method="euclidean")
perm

#loading as "altogether" a file .csv with the name of the species in
the first column (SPECIES) and the principal components in the other
columns

altogether <-read.csv("altogether.csv", sep=";")

#performing the pairwise PERMANOVA

pairwise.adonis <- function(x,factors, sim.function = 'vegdist',
sim.method = 'bray', p.adjust.m ='bonferroni')
{
library(vegan)
co = combn(unique(as.character(factors)),2)
pairs = c()
F.Model =c()
R2 = c()

p.value = c()

for(elem in 1:ncol(co)){
if(sim.function == 'daisy'){
library(cluster); x1 = daisy(x[factors %in%
c(co[1,elem],co[2,elem]),],metric=sim.method)
} else{x1 = vegdist(x[factors %in%
c(co[1,elem],co[2,elem]),],method=sim.method)}
ad = adonis(x1 ~ factors[factors %in% c(co[1,elem],co[2,elem])] );
```

```
pairs = c(pairs,paste(co[1,elem],'vs',co[2,elem]));
F.Model =c(F.Model,ad$aov.tab[1,4]);
R2 = c(R2,ad$aov.tab[1,5]);
p.value = c(p.value,ad$aov.tab[1,6])
}
p.adjusted = p.adjust(p.value,method=p.adjust.m)
sig = c(rep('',length(p.adjusted)))
sig[p.adjusted <= 0.05] <-'.'
sig[p.adjusted <= 0.01] <-'*'
sig[p.adjusted <= 0.001] <-'**'
sig[p.adjusted <= 0.0001] <-'***'
pairw.res = data.frame(pairs,F.Model,R2,p.value,p.adjusted,sig)
print("Signif. codes:  0 ‘***’ 0.001 ‘**’ 0.01 ‘*’ 0.05 ‘.’ 0.1 ‘ ’
1")
return(pairw.res)
}
pairwiseperm <- pairwise.adonis(altogether[,2:8],altogether$SPECIES,
sim.method="euclidean")
pairwiseperm
```

**Consensus (mean) Configuration for *Sparus aurata***

```
XY
1 -0.111985998 0.020825494
2 -0.109615269 0.027619638
3 -0.105202455 0.033560471
4 -0.101604303 0.038974729
5 -0.097894317 0.043416739
6 -0.093670507 0.045761206
7 -0.089495663 0.048886209
8 -0.085697330 0.052428925
9 -0.080969951 0.053887782
10 -0.075346475 0.055932507
11 -0.069737702 0.058770809
12 -0.063997503 0.060441901
13 -0.058144329 0.061274521
14 -0.050867788 0.062236191
15 -0.044108294 0.063687828
16 -0.037899992 0.064872581
17 -0.032046237 0.066164371
18 -0.025519500 0.066979422
19 -0.018893574 0.067832743
20 -0.011352881 0.068824768
21 -0.003379577 0.070184713
22 0.003941716 0.070318777
23 0.010544570 0.069583979
24 0.016163398 0.070575203
25 0.022204114 0.071245986
26 0.028972778 0.069926177
27 0.036306871 0.069272613
28 0.043121591 0.069109615
29 0.049275354 0.067610731
30 0.055725043 0.066267603
```

| | | |
|---|---|---|
| 31 | 0.061325241 | 0.065663669 |
| 32 | 0.066469754 | 0.063617583 |
| 33 | 0.070929592 | 0.061405226 |
| 34 | 0.075949054 | 0.060084953 |
| 35 | 0.081748662 | 0.058161297 |
| 36 | 0.087650502 | 0.056121399 |
| 37 | 0.093681297 | 0.053835021 |
| 38 | 0.099647566 | 0.050511012 |
| 39 | 0.105942531 | 0.046201427 |
| 40 | 0.111627933 | 0.041765006 |
| 41 | 0.117046519 | 0.035927833 |
| 42 | 0.119965934 | 0.030004205 |
| 43 | 0.124317950 | 0.021823507 |
| 44 | 0.126892238 | 0.013682849 |
| 45 | 0.128911015 | 0.004797039 |
| 46 | 0.130613229 | -0.005051971 |
| 47 | 0.131316453 | -0.015659047 |
| 48 | 0.130896179 | -0.026535782 |
| 49 | 0.129275271 | -0.036734035 |
| 50 | 0.126509562 | -0.045688430 |
| 51 | 0.122707948 | -0.053839245 |
| 52 | 0.118385344 | -0.060731195 |
| 53 | 0.113862309 | -0.066369641 |
| 54 | 0.106822967 | -0.072797264 |
| 55 | 0.098386899 | -0.078161369 |
| 56 | 0.088337609 | -0.083553788 |
| 57 | 0.077351304 | -0.088403239 |
| 58 | 0.066259909 | -0.092948633 |
| 59 | 0.055236107 | -0.097312568 |
| 60 | 0.044295903 | -0.100811454 |
| 61 | 0.032439749 | -0.102852482 |
| 62 | 0.019508897 | -0.103627629 |
| 63 | 0.006728570 | -0.103278457 |
| 64 | -0.006111233 | -0.102070086 |
| 65 | -0.018904416 | -0.100520116 |
| 66 | -0.030925212 | -0.098831827 |
| 67 | -0.042678537 | -0.096372740 |
| 68 | -0.053916766 | -0.093145109 |
| 69 | -0.064740943 | -0.089088242 |
| 70 | -0.075500239 | -0.084168973 |
| 71 | -0.085377618 | -0.079033034 |
| 72 | -0.094625680 | -0.073643199 |
| 73 | -0.103541191 | -0.068053624 |
| 74 | -0.111313236 | -0.061587820 |
| 75 | -0.116986813 | -0.054598761 |
| 76 | -0.120370519 | -0.048546017 |
| 77 | -0.122780151 | -0.041843866 |
| 78 | -0.124317713 | -0.034893839 |
| 79 | -0.125148567 | -0.026019525 |
| 80 | -0.124740246 | -0.016192182 |
| 81 | -0.123213700 | -0.006752870 |
| 82 | -0.120800829 | 0.002189001 |
| 83 | -0.118075921 | 0.009538709 |
| 84 | -0.115643921 | 0.014885001 |

85 -0.113617990  0.018346063

**Consensus (mean) Configuration for *Dicentrarchus labrax***

XY
1 -0.084063054 -7.493080e-02
2 -0.085284872 -7.244669e-02
3 -0.085835498 -6.904149e-02
4 -0.086264471 -6.559345e-02
5 -0.086494529 -6.177636e-02
6 -0.086573186 -5.752205e-02
7 -0.086874393 -5.254136e-02
8 -0.086788260 -4.731608e-02
9 -0.086394499 -4.177156e-02
10 -0.086194547 -3.651909e-02
11 -0.085989369 -3.150876e-02
12 -0.085518545 -2.664407e-02
13 -0.084915872 -2.187110e-02
14 -0.084581849 -1.766060e-02
15 -0.084228742 -1.333060e-02
16 -0.083253446 -8.741956e-03
17 -0.082308863 -4.232078e-03
18 -0.081419693 9.900444e-05
19 -0.080668095 4.631907e-03
20 -0.079990814 8.976336e-03
21 -0.078771777 1.314493e-02
22 -0.077787745 1.747186e-02
23 -0.076669813 2.170417e-02
24 -0.074972164 2.571103e-02
25 -0.073165006 2.965923e-02
26 -0.071118542 3.355725e-02
27 -0.069253048 3.789214e-02
28 -0.067101483 4.217630e-02
29 -0.065272117 4.624415e-02
30 -0.063484055 5.035903e-02
31 -0.061086096 5.501976e-02
32 -0.058239352 5.987216e-02
33 -0.055595667 6.455373e-02
34 -0.053206866 6.914893e-02
35 -0.050515808 7.409102e-02
36 -0.047993920 7.861999e-02
37 -0.045838038 8.260369e-02
38 -0.043735897 8.665328e-02
39 -0.041530564 9.066679e-02
40 -0.039193631 9.459754e-02
41 -0.036529720 9.796552e-02
42 -0.033588901 9.989397e-02
43 -0.019540965 9.974978e-02
44 -0.005766037 9.809057e-02
45 0.009201079 9.598398e-02
46 0.025022186 9.330910e-02
47 0.040888691 9.087988e-02
48 0.056368259 8.860723e-02
49 0.071051945 8.628020e-02
50 0.084386090 8.428065e-02
51 0.096293393 8.252662e-02

52  0.107373195  8.035708e-02
53  0.117132257  7.676089e-02
54  0.122551298  6.947517e-02
55  0.126757603  6.055238e-02
56  0.130697172  5.021225e-02
57  0.134495312  3.963552e-02
58  0.137556159  2.849452e-02
59  0.140041130  1.763462e-02
60  0.141778054  6.709850e-03
61  0.142359020 -3.919447e-03
62  0.141656223 -1.346656e-02
63  0.140022991 -2.290315e-02
64  0.137114629 -3.181478e-02
65  0.132967612 -4.067367e-02
66  0.128248995 -4.853831e-02
67  0.122135778 -5.631759e-02
68  0.115476050 -6.376029e-02
69  0.108146591 -7.121215e-02
70  0.100087687 -7.889668e-02
71  0.091544181 -8.641569e-02
72  0.082488754 -9.364631e-02
73  0.073635259 -1.003847e-01
74  0.065415084 -1.062804e-01
75  0.057476391 -1.098208e-01
76  0.044087219 -1.087436e-01
77  0.031300713 -1.053367e-01
78  0.018636517 -1.017244e-01
79  0.005766209 -9.831341e-02
80 -0.007912330 -9.489786e-02
81 -0.021754313 -9.167636e-02
82 -0.036017002 -8.865244e-02
83 -0.049704110 -8.559841e-02
84 -0.062836568 -8.265825e-02
85 -0.074551707 -7.897630e-02

Consensus (mean) Configuration for **Mullus surmuletus**
XY
1 -0.106638874 -0.0633891507
2 -0.110523802 -0.0590257742
3 -0.113433990 -0.0538910719
4 -0.113735367 -0.0479816466
5 -0.110626900 -0.0416636426
6 -0.104846389 -0.0350389310
7 -0.099172698 -0.0293134054
8 -0.095456099 -0.0256073866
9 -0.091946532 -0.0223422279
10 -0.089083842 -0.0199787844
11 -0.088994181 -0.0168839645
12 -0.091014360 -0.0124536589
13 -0.091552845 -0.0065419036
14 -0.087434192 -0.0008441032
15 -0.081369123 0.0043637851
16 -0.078201351 0.0081838393
17 -0.077870504 0.0117949062
18 -0.078683389 0.0156276047

| | | |
|---|---|---|
| 19 | -0.077886566 | 0.0202538116 |
| 20 | -0.073463994 | 0.0254245694 |
| 21 | -0.068123075 | 0.0284764358 |
| 22 | -0.065417557 | 0.0308730278 |
| 23 | -0.063951272 | 0.0340153617 |
| 24 | -0.062887279 | 0.0376900844 |
| 25 | -0.059902742 | 0.0420770913 |
| 26 | -0.055734254 | 0.0458259651 |
| 27 | -0.052961857 | 0.0497418895 |
| 28 | -0.051020997 | 0.0535719077 |
| 29 | -0.046559261 | 0.0561869410 |
| 30 | -0.041188852 | 0.0580318363 |
| 31 | -0.038570509 | 0.0606500533 |
| 32 | -0.037763079 | 0.0657334866 |
| 33 | -0.036933261 | 0.0698776021 |
| 34 | -0.034838585 | 0.0721780280 |
| 35 | -0.032218334 | 0.0727042202 |
| 36 | -0.029731881 | 0.0735291064 |
| 37 | -0.028000284 | 0.0784342295 |
| 38 | -0.027258261 | 0.0877900839 |
| 39 | -0.025022808 | 0.0981799992 |
| 40 | -0.020283030 | 0.1047184972 |
| 41 | -0.013984992 | 0.1069964445 |
| 42 | -0.006495916 | 0.1070137794 |
| 43 | 0.012027381 | 0.1032354827 |
| 44 | 0.027590449 | 0.0990187704 |
| 45 | 0.042401522 | 0.0947478533 |
| 46 | 0.057516896 | 0.0894981641 |
| 47 | 0.071832707 | 0.0832159986 |
| 48 | 0.084907954 | 0.0764512130 |
| 49 | 0.096466076 | 0.0695654110 |
| 50 | 0.106405886 | 0.0632435102 |
| 51 | 0.113885045 | 0.0581104779 |
| 52 | 0.120125777 | 0.0534457006 |
| 53 | 0.125936318 | 0.0477787121 |
| 54 | 0.130700467 | 0.0402520598 |
| 55 | 0.134167584 | 0.0324047091 |
| 56 | 0.136320642 | 0.0239394540 |
| 57 | 0.137737600 | 0.0150379473 |
| 58 | 0.138304667 | 0.0051416234 |
| 59 | 0.138557803 | -0.0053393558 |
| 60 | 0.138316884 | -0.0154192993 |
| 61 | 0.137412991 | -0.0247019142 |
| 62 | 0.135941668 | -0.0329453123 |
| 63 | 0.133690906 | -0.0414034838 |
| 64 | 0.129992599 | -0.0495768418 |
| 65 | 0.122974774 | -0.0579267912 |
| 66 | 0.115869218 | -0.0641321904 |
| 67 | 0.108910359 | -0.0697305651 |
| 68 | 0.101829910 | -0.0748668261 |
| 69 | 0.093816620 | -0.0803541709 |
| 70 | 0.085111137 | -0.0861402071 |
| 71 | 0.076136708 | -0.0917236438 |
| 72 | 0.066919068 | -0.0973075105 |
| 73 | 0.057512300 | -0.1018984126 |

| | | |
|---|---|---|
| 74 | 0.047946644 | -0.1055100467 |
| 75 | 0.038216710 | -0.1073277042 |
| 76 | 0.024098468 | -0.1083350965 |
| 77 | 0.009573835 | -0.1091085718 |
| 78 | -0.004644525 | -0.1089124421 |
| 79 | -0.019220490 | -0.1072421659 |
| 80 | -0.033763710 | -0.1037176747 |
| 81 | -0.048134189 | -0.0985336525 |
| 82 | -0.061710235 | -0.0922347295 |
| 83 | -0.074770066 | -0.0848747540 |
| 84 | -0.087622052 | -0.0771221981 |
| 85 | -0.099968005 | -0.0686327779 |
